# Overcoming Leverage in Superior Dentures

**Published:** 1887-04

**Authors:** L. C. Bryan

**Affiliations:** Basel, Switzerland


### OVERCOMING LEVERAGE IN SUPERIOR DENTURES.

BY L. C. BRYAN, BASEL, SWITZERLAND.

There is a large class of cases applying for artificial dentures, which give the operator serious and continued trouble in their adjustment to retain their hold on the membrane, under the strain brought to bear on them from the remaining lower incisors, or incisors and bicuspids. The patient presents an edentulous superior maxillary, with from six to ten lower front teeth and no remaining molars. In many cases it is impossible to persuade the patient to have a lower set to provide a masticating surface at the back, which would equalize the strain and give an adjustment of the articulation which would produce the desired result. Often if a lower set is made, the patient cannot or will not wear them, and the result is the same; the upper denture is tilted down at the back, food works under and discomfort results, even if the case does not come completely out. These lower front teeth continue to displace the most carefully adjusted upper dentures, and the inventive skill of the dentist is taxed to its utmost, in most cases without satisfactory results. The operator, in despair, finally dismisses the case, with the assurance that the only help for the unhappy patient lies in patient practice and experimental manipulation of food in mastication until sufficient control is acquired.

A case of this kind which the writer has treated has given such comfort to the wearer and relief to himself, that the method of overcoming the difficulty is here presented. The first step was to secure a perfect adaptation of the upper set. A loose impression in modeling composition was taken in a long cup extending over the tuberosities, and a model was made over which an impression cup of soft sheet tin was loosely fitted. Sheet lead would serve the same purpose. There is a composition largely advertised in Europe, which is simply shellac rolled into sheets, bronzed over and ornamented with its title in raised letters. Shellac may be rolled out, and when dipped in hot water can be readily pressed into any desired form of impression cup, and although somewhat brittle if too thin, it serves the purpose in an admirable manner.*

A cup being fitted loosely to the model, slow setting plaster was mixed, salted and stirred, until it began to stiffen and could be built in a pyramid in the center of the cup, so that when pressed up no air bubbles would remain in the palatal portion. A small quantity of plaster was placed with a teaspoon handle on the buccal side of each tuberosity and the cup pressed carefully up, first posteriorly, cutting off the flow of plaster backward and causing the surplus to press out labially. When firmly set the impression was found to have a strong suction hold, which is the first element of success. The resulting model was heroically trimmed labially and buccally (to compensate for the expansion of the plaster model), and also on the soft parts palatally. Folsom ridges were also cut across the posterior part of the model with a spoon-bill excavator, and a long, narrow air-chamber adjusted over the hard ridge of the palate. The bite being secured and the teeth adjusted, a sufficiency of wax was built down at the tuberosities to articulate with the ridge of the lower jaw for half inch in length.

When packing, a thin layer of red rubber, of which the plate was composed, was carried to the end and over the tuberosities, and the remainder of the bite built down with velum or soft vulcanizable rubber, somewhat higher than the wax bite indicated. The result was all that was anticipated. This soft cushion at the back gave no pain to the lower ridge, and held the case firmly up at the back while masticating on the front teeth.

Presumably the last molars of the upper set might be elongated to articulate with the lower alveolar ridge, or the more durable hard rubber be extended down for the same purpose, but the soft rubber cushion gives no pain to tender lower ridges of old persons, and can be elongated to touch slightly before the incisors meet, thus anticipating the leverage.

---

*An ordinary britannia impression cup may be bent to an approximate fit, and in this a wax impression taken, which can be carved to an exact adaptation for the plaster impression. The wax should be melted upon the cup sufficiently to make it adhere.—Editor.

